# New Corticopontine Connections in the Primate Brain: Contralateral Projections From the Arm/Hand Area of the Precentral Motor Region

**DOI:** 10.3389/fnana.2018.00068

**Published:** 2018-08-17

**Authors:** Robert J. Morecraft, Jizhi Ge, Kimberly S. Stilwell-Morecraft, Diane L. Rotella, Marc A. Pizzimenti, Warren G. Darling

**Affiliations:** ^1^Division of Basic Biomedical Sciences, Laboratory of Neurological Sciences, Sanford School of Medicine, The University of South Dakota, Vermillion, SD, United States; ^2^Department of Health and Human Physiology, Motor Control Laboratories, The University of Iowa, Iowa City, IA, United States; ^3^Department of Anatomy and Cell Biology, Carver College of Medicine, The University of Iowa, Iowa City, IA, United States

**Keywords:** pyramidal tract, frontal lobe, corticofugal, pons, cerebrocebellar, cerebellum, hand coordination

## Abstract

The ipsilateral corticopontine projection (iCPP) represents a massive descending axon system terminating in the pontine nuclei (PN). In the primate, this projection is well known for its dominant influence on contralateral upper limb movements through the classical cerebrocerebellar circuity system. Although a much weaker contralateral corticopontine projection (cCPP) from motor cortex to the paramedian region has been reported in the non-human primate brain, we provide the first comprehensive description of the cCPP from the lateral motor cortex using high resolution anterograde tract tracing in *Macaca mulatta*. We found a relatively light cCPP from the hand/arm area of the primary motor cortex (M1), comparatively moderate cCPP from ventrolateral premotor cortex (LPMCv) and a more robust and widespread cCPP from the dorsolateral premotor cortex (LPMCd) that involved all nine contralateral PN. The M1 projection primarily targeted the dorsal pontine region, the LPMCv projection targeted the medial pontine region and LPMCd targeted both regions. These results show the first stage of the primate frontomotor cerebrocerebellar projection is bilateral, and may affect both ipsilateral and contralateral limbs. Clinically, the cCPP originating in the non-injured hemisphere may influence the recovery process of the more affected upper extremity following subtotal unilateral damage to the lateral cortical region. The cCPP may also contribute to the mild impairment of the upper limb contralateral to a unilateral cerebellar injury.

## Introduction

**Figure 1:**
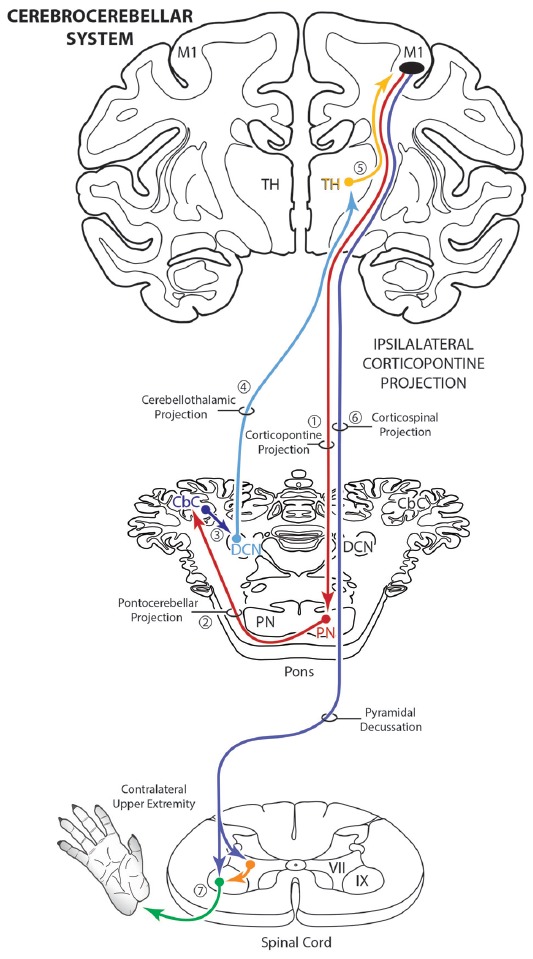
Schematic diagram illustrating the classical cerebrocebellar circuit originating from the primary motor cortex (M1). The sequential stages of the circuit are numbered starting with the powerful descending ipsilateral corticopontine projection (iCPP) to the pontine nuclei (PN) (1). Note that the PN project to the contralateral cerebellar cortex (CbC) (2) and the major cerebellar output from the deep cerebellar nuclei (DCN) ascends to innervate the contralateral thalamus (TH) (4). The thalamus then projects back to the ipsilateral motor cortex (5). The final effect of this cerebrocebellar circuit is on the contralateral upper extremity, via the nearly exclusive contralateral M1 corticospinal projection (CSP) to spinal interneurons (orange) and motoneurons (green) (7). Not illustrated is a small ipsilateral CSP from M1 that represents only 2% of the total terminal bouton CSP with 98% representing the contralateral terminal CSP (Morecraft et al., 2013).

The corticopontine projection from motor cortex represents a major descending axon system in the primate brain that terminates in an expansive brainstem region occupied by the pontine nuclei (PN). For decades, this projection has been of central interest to neuroscientists studying motor behavior and in particular, corticocerebellar influence on the control of movement. This is largely due to the robust ipsilateral corticopontine projection (iCPP) originating from primary motor (M1) and lateral premotor (LPMC) cortical regions (Brodal, 1978a; Glickstein et al., 1985) which are well known for their dominant influence on mediating upper limb movements contralateral to the precentral motor cortex. This behavioral outcome largely occurs as a result of the underlying anatomy (Figure 1), consisting of a crossed pathway from the ipsilateral PN to the contralateral cerebellum followed by a crossed ascending pathway passing from the cerebellum to the contralateral thalamus and eventually back to motor cortex from which the iCPP originated. The final functional effect is achieved through the predominantly contralateral corticospinal projection (CSP) arising from the precentral motor cortex.

Interestingly, a contralateral corticopontine projection (cCPP) from motor cortex has been experimentally demonstrated in a number of animal models. In the rodent, the cCPP is much weaker than the iCPP and the primary target appears to be the ventral, ventromedial and medial pontine regions (Mihailoff et al., 1985; Leergaard and Bjaalie, 2007) but its functional contribution remains unclear. In the non-human primate (*Macaca fascicularis*), the cCPP from motor cortex is also weaker than the extraordinarily powerful iCPP, and has been described as occupying the paramedian region of the basis pontis (Hartmann-von Monakow et al., 1981). Despite the uncertainties concerning function of the cCPP, when considering the overall laterality of the classical cerebrocerebellar system, one would suspect that the major functional effect of a cCPP would be to mediate control of the upper extremity ipsilateral to the motor cortex from which the circuit began. That is, because of the crossed cerebello-thalamo-cortical ascending feedback loop, the cCPP would terminate in motor cortex located in the opposite hemisphere from where it originated. In addition to a potential role in bilateral limb movements, this raises the possibility that a precentral cCPP from the non-lesioned hemisphere following unilateral stroke could influence recovery of the more affected limb located ipsilateral to the non-lesioned hemisphere.

It is important to consider that the corticopontine projection from the primary motor and lateral premotor cortices has been the subject of numerous neuroanatomical reports in the monkey (Dhanarajan et al., 1977; Brodal, 1978a; Künzle, 1978; Wiesendanger et al., 1979; Hartmann-von Monakow et al., 1981; Glickstein et al., 1985; Leichnetz, 1986; Schmahmann et al., 2004). Notably, this entire body of work relied on the most sensitive tract tracing methods available at the time of the conducted experiments. Namely, nerve axon and terminal degeneration tracing methods for the earliest studies, to the use of horseradish peroxidase (HRP) and tritiated amino acids (H^3^AA) for the relatively more contemporary reports. Despite the significant contribution of this collection of seminal work, it is important to recognize that a major limitation of these neuroanatomical tracing methods as determined at the light microscopic level is that the applied histochemical procedures produce background levels of artifact that compete with positively labeled axons and axon terminals (Morecraft et al., 2014). Consequently, it is difficult to identify relatively lighter projection fields with confidence, and in particular, labeling that is dispersed in the neuropil. Furthermore, distal portions of individual axons and affiliated terminal boutons cannot be evaluated with these tract tracing methods. Given these technical limitations, we reinvestigated the issue of a potential cCPP in the intact primate brain by utilizing the highly sensitive dextran anterograde tract tracer method in combination with immunohistochemistry following tracer injection into different parts of the lateral frontal cortex involved in mediating arm/hand motor control. Using an unbiased stereology approach, we sought to determine whether the cCPP from the precentral and premotor motor regions constitutes a consistent projection system in the PN. A second goal was to quantitatively determine the relative strength of the projections (i.e., total bouton number) to the individual basilar PN from the precentral motor and premotor cortices.

## Materials and Methods

**Figure 2:**
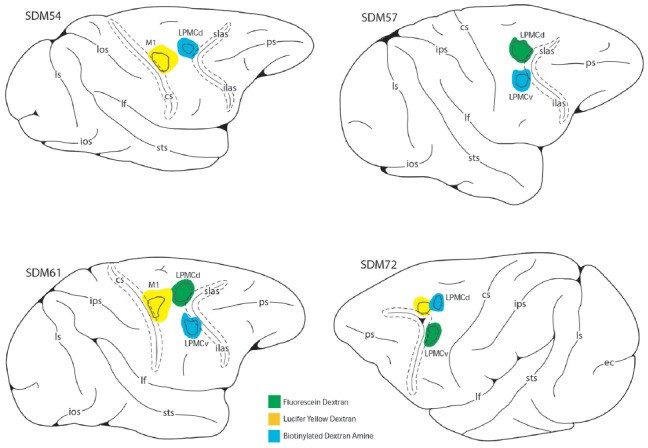
Line drawings of the lateral surface of the cerebral cortex in cases SDM54, SDM57, SDM61 and SDM72 depicting the respective lucifer yellow dextran (LYD), fluorescein dextran (FD) and biotinylated dextran amine (BDA) injection sites into the cortical hand/arm region of M1, caudal part of LPMCd and dorsal part of LPMCv. On the color-coded injection sites, the irregular shaped black line within the injection site represents the boundary between the centrally located injection site core, and peripherally located injection site halo. All injection sites were localized using intracortical microstimulation (ICMS; Morecraft et al., 2007a, 2013).

To accomplish this study seven monkeys (*Macaca mulatta*) were used. Contemporary experimental tract tracing procedures were applied in the central nervous system of four monkeys (Figure 2; Table 1). The tracer injections were located in the arm/hand region of M1, the caudal region of the dorsolateral premotor cortex (LPMCd) and dorsal region of ventrolateral premotor cortex (LPMCv; Figure 2; Table 1) and terminal bouton labeling was assessed in the contralateral pontine gray matter from 10 injection sites. Of these tracer experiments, six injection sites were evaluated quantitatively using stereology (Table 1). We also investigated the anatomical pathway/route by which the cCPP gains access to the contralateral PN because this information may be useful for designing human tractography studies that focus on mapping projections of the cerebrocebellar circuit (e.g., Palesi et al., 2017; Schulz et al., 2017). Finally, three additional animals were used for cytoarchitectonic analysis of pontine gray matter organization using NeuN stained tissue sections through the pons. All experimental protocols were approved by The University of South Dakota Institutional Animal Care and Use Committee. All phases of this study were conducted at The University of South Dakota in accordance with United States Department of Agriculture (USDA), National Institutes of Health and Society for Neuroscience guidelines for the ethical treatment of animals. All monkeys were housed and cared for in a USDA and AALAC approved and inspected facility.

**Table 1:** Description of the experimental parameters in each case.

Case	Sex	Age (years)	Weight (kg)	Area injected	Tracer/Injections	Total volume (μL)	Injection core vol. (mm^3^)	Injection halo vol. (mm^3^)	Post-injection survival (days)
SDM54	Male	9.0	9.2	M1 arm	LYD/3*	1.2	10.66	64.7	33
				LPMCd	BDA/3	1.2	15.44	78.44
SDM57	Female	18.0	6.0	LPMCd	FD/3*	1.2	15.33	78.44	32
				LPMCv	BDA/3*	1.2	4.01	21.43
SDM61	Female	4.0	4.3	M1 arm	LYD/3*	1.2	13.19	85.50	33
				LPMCd	FD/3*	1.2	20.82	96.55	
				LPMCv	BDA/3	1.2	5.47	28.07	
SDM72	Female	8.7	5.6	LPMCv	FD/3*	1.2	7.16	90.32	33
				LPMCd	BDA/3	1.2	3.56	25.06
				LPMCd	LYD/3	1.2	7.88	52.42	

*Performed stereological estimate of bouton number.

### Neurosurgery and High-Resolution Dextran Tract Tracing Procedures

Preoperatively, each monkey was immobilized with atropine (0.5 mg/kg) then ketamine hydrochloride (10 mg/kg). Each subject was intubated, placed on a mechanical ventilator and anesthetized with a mixture of 1.0%–1.5% isoflurane and surgical grade air/oxygen. Once deeply anesthetized, each animal was placed in a neurosurgical head holder and mannitol was administered intravenously (1.0–1.5 g/kg) to reduce overall cortical volume and enhance surgical accessibility of the brain. Under sterile conditions and isofluorane anesthesia, a skin flap was made over the cranium followed by an oval bone flap positioned over the precentral cortical region (Morecraft et al., 2007a, 2013; McNeal et al., 2010). In all cases the precentral region, extending from the central sulcus to the cortex forming the arcuate sulcus was mapped using intracortical microstimulation (ICMS) as previously described (McNeal et al., 2010; Morecraft et al., 2013). After ICMS mapping, the anterograde neural tract tracer lucifer yellow dextran (LYD), fluorescein dextran (FD), or biotinylated dextran amine (BDA; Molecular Probes, Eugene, OR, USA) was injected into the central region of the physiologically localized arm representation of M1 or directly within a region in LPMCd or LPMCv surrounded by well-defined upper extremity motor responses. Graded pressure injections with a Hamilton microsyringe were made approximately 3–4 mm (for M1) and 2–3 mm (for LPMC) below the pial surface (Table 1). The craniotomy was closed as described previously (McNeal et al., 2010; Morecraft et al., 2013). Penicillin (procaine G) injected IM was used as a pre- and post-surgical antibiotic.

### Tissue Processing and Immunohistochemical Procedures

**Figure 3:**
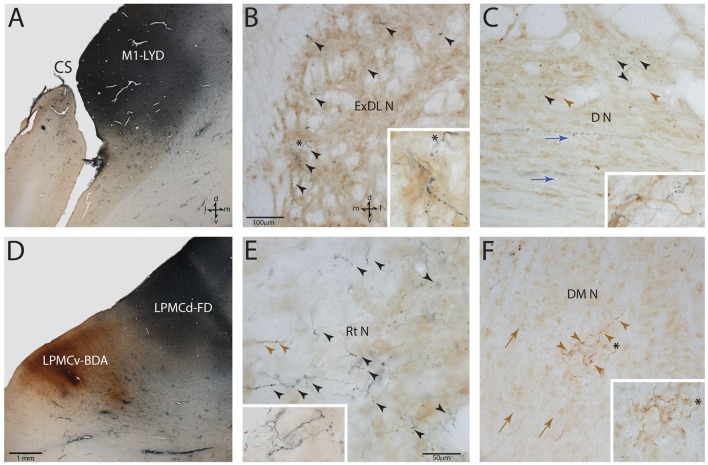
Photomicrographic montage of representative examples of lateral motor area injection sites and labeled boutons in the contralateral PN taken from immunohistochemically developed tissue sections under brightfield microscopic illumination. **(A)** Coronal section through the LYD injection site (blue reaction product) placed in the arm representation of M1 in case SDM54. The anatomical orientation (bottom right) of this panel also applies to panel **(D)**. **(B)** Transverse section through inferior levels of the basis pontis in case SDM54 showing LYD labeled fibers and terminals (black arrowheads) in the contralateral extreme dorsolateral nucleus (ExDL N) following the LYD injection in the M1 arm/hand representation. The inset is a higher power micrograph from the terminal field in the main panel marked by the black asterisk. The anatomical orientation (bottom right) of this panel also applies to panels **(C,E,F)**. The micron bar (bottom left) applies to panels **(C,F)**. **(C)** Transverse section through lower levels of the pons in case SDM54 showing labeled terminals in the dorsal nucleus (D N) following an injection of LYD into M1 (blue reaction product and black arrow heads) and BDA into LPMCd (brown reaction product and brown arrowheads). The inset is from a different focal plane of the same section showing a BDA labeled fiber (brown) en passant, and LYD labeled terminal boutons (blue). The blue arrows show the pathway direction of labeled fibers passing through the dorsal nucleus en route to the more laterally located dorsal tier nuclei (i.e., to the dorsal lateral nucleus and extreme dorsolateral nucleus). **(D)** Low power image depicting the FD (blue reaction product) and BDA (brown reaction product) injection sites in LPMCd and LPMCv, respectively in case SDM57. The calibration bar in the bottom left of the panel also applies to panel **(A)**. **(E)** Transverse section though mid-levels of the pontine gray matter in case SDM57 showing BDA terminal boutons (brown arrowheads) and LYD labeled terminal boutons (black arrowheads) in the contralateral reticular nucleus (Rt N). The inset is from a different focal plane of the same tissue section showing a terminal cluster of LYD labeled axons and terminals. **(F)** Transverse section through mid-levels of the basis pontis in case SDM57 showing BDA labeled terminals (brown arrowheads) in the contralateral dorsal medial nucleus (DM N). The inset is a higher power micrograph from the terminal field in the main panel marked by the black asterisk. The brown arrows show the dorsolateral trajectory of labeled fibers passing through the dorsomedial nucleus en route to the laterally located dorsal tier nuclei (i.e., dorsal nucleus, dorsal lateral nucleus and extreme dorsolateral nucleus). Abbreviations: cs, central sulcus; d, dorsal; l, lateral; LPMCd, dorsolateral premotor cortex; LPMCv, ventrolateral premotor cortex; m, medial; M1, primary motor cortex; v, ventral. For other abbreviations see Figure 2.

Following the survival period after tract tracer injection (Table 1), each monkey was deeply anesthetized with an IP delivered overdose of pentobarbital (50 mg/kg or more) and perfused transcardially with 0.9% saline followed by 4% paraformaldehyde and sucrose (McNeal et al., 2010; Morecraft et al., 2013). All solutions were prepared in 0.1 M phosphate buffer at a pH of 7.4. The cortex was frozen sectioned at 50 μm thickness in the coronal plane. The brainstem was sectioned at the same thickness but in the transverse plane (i.e., 90° angle to the long axis of the brainstem). The tissue sections were collected in cycles of 10 and stored in 4°C. Two adjacent series of tissue sections were processed with the first using a single labeling procedure for visualization of BDA and the second series of tissue sections for double labeling of two injected neural tracers (e.g., BDA and LYD) as previously described (Morecraft et al., 2007a; Figure 3). One additional series of cortical and brainstem tissue sections was stained for Nissl substance for cytoarchitectural analysis using thionin (Morecraft et al., 1992). In three additional animals, NeuN stained sections were prepared through the brainstem using immunohistochemistry as previously described (Morecraft et al., 2012, 2013, 2015b). The NeuN sections were used for cytoarchitectonic verification of the PN boundaries as discerned using the Nissl stained tissue sections processed for each individual tract tracing experiment.

### Anatomical Nomenclature of the Pontine Nuclei

**Figure 4:**
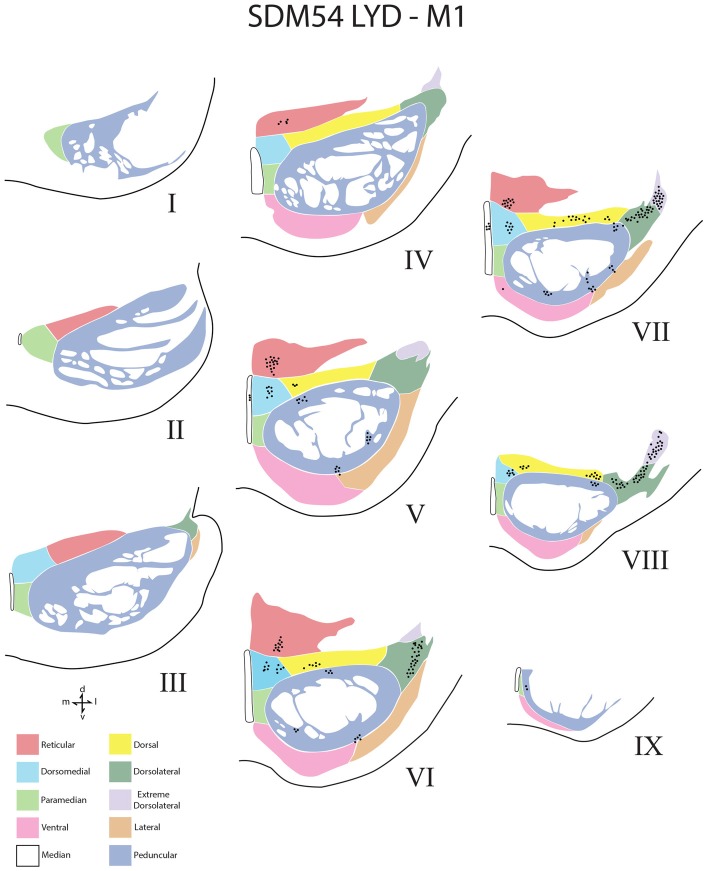
Line drawings of transverse sections depicting the topographical distribution of labeled terminals (black dots) from superior (I) to inferior (IX) levels of the basis pontis in case SDM54 following an injection of LYD into the arm/hand region of M1. Note terminal labeling is primarily located in the dorsal region of the PN and at middle and inferior pontine levels. Each nucleus is identified by the color coded legend in the bottom left of the figure. The descending fibers of the longitudinal pontine fasciculus (LPF) are located in the central region of the peduncular nucleus. Abbreviations: d, dorsal; l, lateral; m, medial; v, ventral.

**Figure 5:**
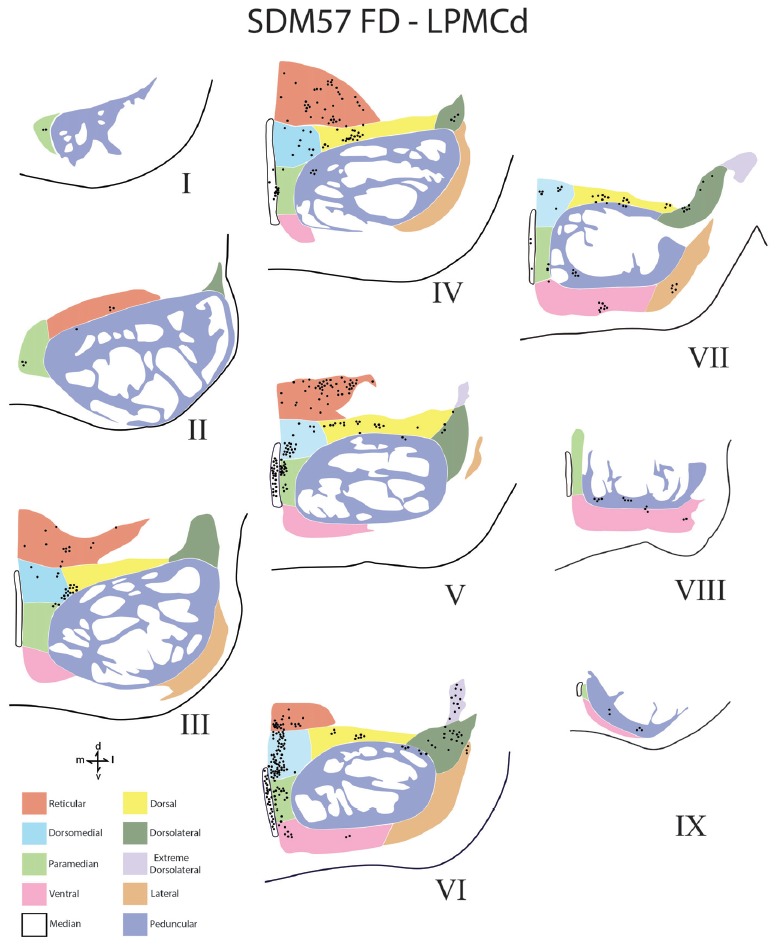
Line drawings of transverse sections depicting the topographical distribution of labeled terminals (black dots) from superior (I) to inferior (IX) levels of the basis pontis in case SDM57 following an injection of FD into the caudal region of LPMCd. Note terminal labeling is primarily located in the dorsal and medial regions of the basis pontis and is particularly prominent at levels IV through VII. Abbreviations: d, dorsal; l, lateral; m, medial; v, ventral.

**Figure 6:**
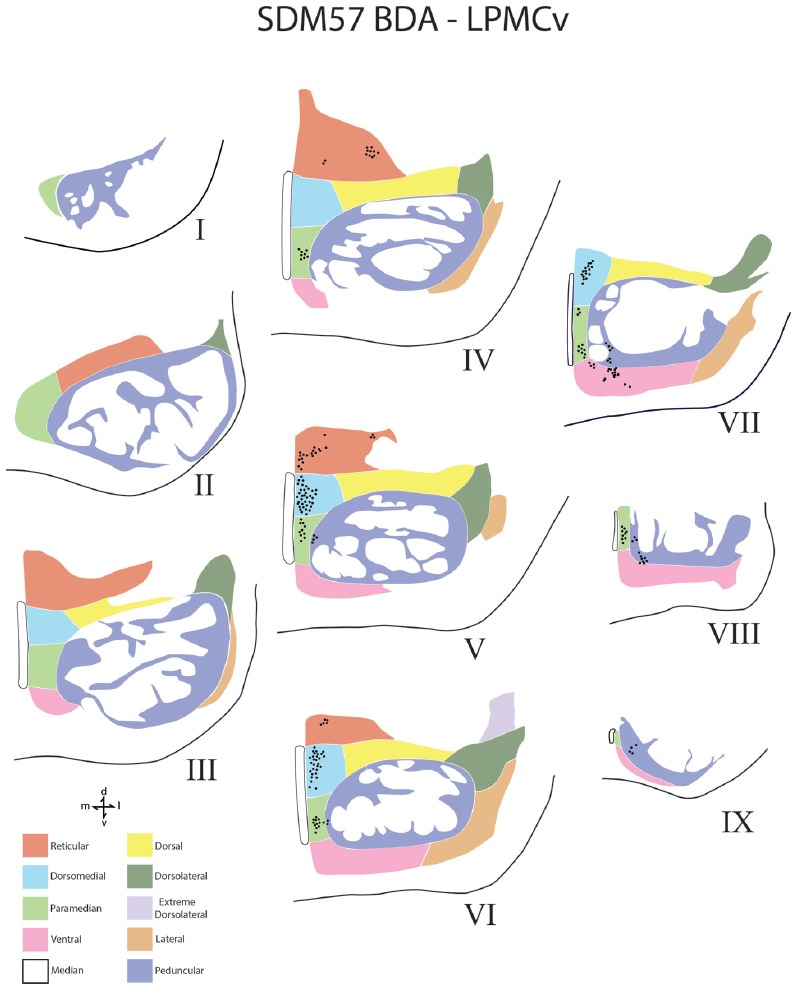
Line drawings of transverse sections depicting the topographical distribution of labeled terminals (black dots) from superior (I) to inferior (IX) levels of the basis pontis in case SDM57 following an injection of BDA into the dorsal region of LPMCv. Note terminal labeling is nearly exclusively located in the medial pontine region and at middle and inferior pontine levels. Abbreviations: d, dorsal; l, lateral; m, medial; v, ventral.

The contralateral distribution of terminal axon labeling was studied in the basis pontis, which is located ventral to the tegmental region of the pons (Afifi and Bergman, 1980). This region was subdivided into nine anatomically affiliated nuclei and a single midline, or median nucleus according to the descriptions of Nyby and Jansen (1951) and the modifications of their subdivisions adopted by Schmahmann and Pandya (1989); Nyby and Jansen (1951); Figures 4–6). Briefly, the nuclei are termed for the most part by their general anatomical location, regional cellular characteristics and occasionally cell-sparse borders as defined using both Nissl and NeuN staining methods. Thus, in the dorsal basilar region dorsal, dorsal medial, dorsal lateral and dorsolateral extreme nuclei were localized. Along the pontine midline, median and paramedian nuclei were recognized. Ventrally, ventral and lateral nuclei were acknowledged and the intrapeduncular nucleus was identified in the central basilar region. Finally, the projection to the reticular nucleus or, nucleus reticularis tegmenti pontis (NRTP; Brodal, 1980) was included in our analysis. Unlike the recognition of a peripeduncular region and a centrally located peduncular region by Schmahmann and Pandya, we considered both of these regions as a single peduncular nucleus for stereological assessment. In addition, terminal labeling was considered using nine rostrocaudal levels of the pons as originally recognized by Nyby and Jansen (1951) and the general anatomical recognition of upper, middle and lower levels as reported by Schmahmann and colleagues (Schmahmann and Pandya, 1989, 1991, 1997a; Schmahmann et al., 2004).

### Stereological Analysis

Terminal bouton number in the contralateral and ipsilateral PN from M1, LPMCd and LPMCv was estimated using brightfield microscopy and stereological counting methods (Glaser et al., 2007; West, 2012). For the current report, the contralateral projection is presented. The methods used to calculate unbiased estimated bouton numbers within PN are the same as those described for quantifying bouton number in the spinal cord and amygdala which have been described in detail in our previous articles (Morecraft et al., 2007b, 2013; McNeal et al., 2010). Estimates of the total number of terminal boutons within each pontine nucleus was determined using a 100× oil objective, the Optical Fractionator Probe and our Neurolucida equipped microscope workstations (Microbrightfield, Colchester, VT, USA). For the current project the counting frame measured 100 μm × 70 μm and spacing interval between counting frames was 300 μm × 200 μm. Every other pontine tissue section was used for stereological evaluation totaling 7–9 sections in a given animal. Total number of boutons were estimated in each nucleus. We did not stereologically quantify boutons in the upper, middle and inferior levels (e.g., from a rostral to caudal perspective) because the nuclei are not present in all levels (only the median and peduncular nuclei are typically present at most levels). To address this issue, density plots were created of all nine pontine levels in each case and reported qualitatively (Figures 4–6).

Estimates of the tracer injection site volumes (which included the halo volume and core volume) were also calculated (Table 1). Specifically, injection site volumes were determined using the Cavalieri probe and same StereoInvestigator software as described in our prior work (Pizzimenti et al., 2007; Morecraft et al., 2015a, 2016). The core area of the injection site is viewed as the primary uptake/transport region of the tract tracer injection site (Mesulam, 1982; Condé, 1987).

## Results

**Figure 7:**
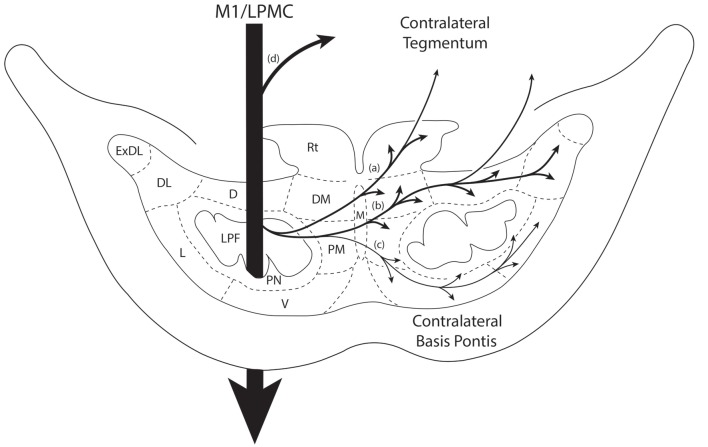
Summary diagram illustrating the main pathways taken by labeled axons to innervate the gray matter of the contralateral basis pontis. The thick descending arrow denotes the LPF. One major route emerges from the LPF and courses dorsally through the median nucleus (a,b) to innervate the dorsomedial, reticular, dorsal, dorsolateral and dorsolateral extreme PN. This pathway also contains a contingent of fibers that continue dorsally through the contralateral basis pontis to enter and innervate gray matter targets in the contralateral pontine tegmentum. Injections in the lateral premotor cortex (LPMC) also gave rise to a weaker, ventrally directed pathway (c). Also illustrated is the commonly recognized route taken by fibers from the LPF that innervate the contralateral pontine tegmentum (d). Specifically these fibers emerge from the LPF, enter the ipsilateral tegmentum, then cross the midline within the tegmentum proper. Abbreviations: D, dorsal nucleus; DL, dorsolateral nucleus; DM, dorsomedial nucleus; ExDL, extreme dorsolateral nucleus; L, lateral nucleus; M, median nucleus; PM, paramedian nucleus; PN, peduncular nucleus; Rt, reticular nucleus; V, ventral nucleus.

In all experimental cases a cCPP was found. We examined labeled fibers in the midbrain crus cerebri (of the cerebral peduncle) and longitudinal pontine fasciculus (LPF) in the pons (e.g., the vertically descending corticofugal/pyramidal tract through the central region of the right and left basis pontis), for the presence of labeled fibers to discern the general course of the descending pathway by which labeled fibers reached the contralateral PN. The crus cerebri is located immediately above the pons and represents an anatomically isolated white matter fascicle appropriate for evaluating descending cortical efferent fiber system laterality in the upper brainstem. In all four monkey cases (i.e., 10 injection sites), descending labeled axons were exclusively found in the centromedial region of the ipsilateral midbrain cerebral peduncle as previously reported for the M1, LPMCd and LPMCv pathways (Morecraft et al., 2007a). Likewise, labeled axons were solely located in the ipsilateral LPF. These findings indicate that the cCPP fibers descended in the ipsilateral corticofugal/bulbar tract and then crossed the midline within the basis pontis to innervate contralateral PN. This route was further validated by observing dextran labeled axons leaving the ipsilateral LPF to cross the midline by passing through the ipsilateral median, paramedian, dorsomedial nuclear regions (Figure 7). Once within the contralateral basis pontis, axons coursed either dorsal or ventral to the centrally located LPF (Figures 3F, 7). Rarely did a labeled axon pass through the medial corner of the contralateral LPF. No labeled axons were found in the contralateral (or ipsilateral) middle cerebellar peduncle, thereby demonstrating contralateral labeled fibers were not a result of transsynaptic labeling from cell bodies located in the ipsilateral pontine gray matter.

**Figure 8:**
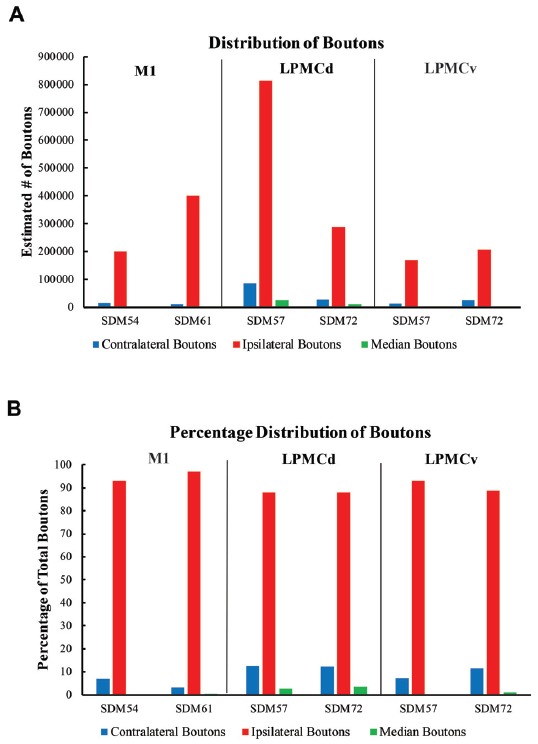
**(A)** Bar graphs illustrating the total estimated number of contralateral corticopontine boutons and ipsilateral corticopontine boutons following injections of high resolution tract tracer into M1, LPMCd and LPMCv in each experimental case. **(B)** Bar graphs illustrating the percent distribution of the contralateral corticopontine boutons and ipsilateral corticopontine boutons following injections of high resolution tract tracer into M1, LPMCd and LPMCv in each experimental case.

**Figure 9:**
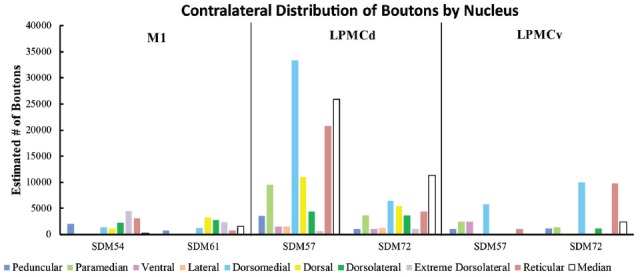
Estimated number of contralateral corticopontine boutons within each pontine nucleus for each injection site in M1, LPMCd and LPMCv in each experimental case.

In general, we found a light cCPP from the M1, a relatively moderate cCPP from LPMCv, and a more robust cCPP from the LPMCd. A reoccurring characteristic of the cCPP included light patches of small terminal clusters scattered throughout the nuclei, with some labeled boutons located on single, isolated axon terminals (Figures 3B,C,F). Although not the focus of the present report, the iCPP in all cases was substantially greater than the cCPP (Figure 8). However, there were consistent contralateral patterns of terminal labeling noted following tract tracer injection into the respective motor areas prompting further investigation (Figure 9). Below we describe the cCPP from all three lateral motor areas.

### Contralateral Pontine Labeling From M1

A small subset of M1 fibers emerged from the heavily labeled ipsilateral LPF, directed their trajectory medially and crossed the midline. Some fibers abruptly terminated in the median, contralateral paramedian and contralateral peduncular nuclei, whereas other labeled fibers arched dorsally over the dorsomedial corner of the contralateral LPF passing into the general region of the dorsal tier nuclei and dorsal part of the peduncular nucleus (Figures 7a,b). At this location, fibers coursed over the dorsal edge of the contralateral LPF. Once within the dorsal tier nuclear region fibers continued to course laterally to innervate dorsal PN (Figures 3C, 7a,b). It is noteworthy that a subset of M1 fibers, which coursed dorsally, eventually passed through the basis pontis to enter the contralateral pontine tegmentum to innervate gray matter structures in this location (e.g., contralateral pontine reticular formation; Figures 7a,b).

Overall, the M1 terminal projection was light and primarily targeted the contralateral dorsal pontine gray matter at middle to inferior pontine levels (Figures 3B,C, 4, 9). There was no evidence of labeled fibers or terminals in the upper third of the contralateral basis pontis (Figure 4). The dorsomedial nucleus, dorsal nucleus, dorsolateral and extreme dorsolateral nucleus were consistently labeled with small, isolated patches of terminal boutons. The medial region of the reticular nucleus also had terminal labeling. In addition, some diffuse labeling was found in the peduncular nucleus and median nucleus. Finally, terminal labeling was not found in the paramedian, ventral or lateral nuclei following injection of dextran tracer into the hand/arm region of M1.

### Contralateral Pontine Labeling From LPMCd

Labeled fibers from LPMCd emerged from the ipsilateral LPF and some coursed medially to terminate in the median nucleus while others crossed the midline to innervate the contralateral paramedian nucleus and adjacent (medial) part of the contralateral peripedunclar nucleus. However, a large number of labeled fibers from LPMCd, after passing through the midline region, arched dorsally over the dorsomedial corner of the longitudinal pontine tract to enter the general region of the dorsal tier nuclei (Figures 7a,b). Very few fibers were found to pass through the territory occupied by the contralateral LPF. As with M1 fibers, some passed through the contralateral basis pontis to enter the contralateral pontine tegmentum (Figures 7a,b), whereas other fibers continued to course laterally within the dorsal basis pontis region (Figures 3C, 7b) to innervate the dorsal group of nuclei with some fibers peeling off to innervate the dorsal region of the peduncular nuclei. From the ipsilateral LPF a few LPMCd labeled fibers also curved ventrally across the midline to enter the contralateral ventral pontine gray matter region, curving around the ventromedial corner of the LPF, then laterally through the ventral tier nuclei with some fibers peeling off to innervate the ventral region of the peduncular nuclei (Figure 7c). The relatively heavy distribution of dorsally located fibers, which included the fibers *en passant* to the contralateral tegmentum, corresponded with the heavier distribution of bouton labeling in the dorsal tier nuclei as described below. In comparison, the lighter distribution of ventral fibers coincided with the relatively lighter density of terminal boutons ventrally, also described below.

Contralateral pontine labeling from LPMCd was much heavier and widespread compared to the M1 projection as well as the LPMCv projection (Figure 5). In general, case SDM57 FD gave rise to the strongest cCPP of all cases studied (Figure 9). This was exemplified by the finding of labeled boutons involving all pontine levels (upper, middle and lower). However, very light labeling was found at levels I and II as well as VIII and IX, with more robust labeling at levels III-VII (Figure 5). Although terminal labeling was preferentially located in the dorsal and medial pontine region, evidence of tracer-filled boutons was found in all nine contralateral PN, in addition to a strong projection to the median nucleus (Figure 9). Specifically, significant pontine labeling occurred within the reticular nucleus and neighboring dorsomedial, dorsal and dorsolateral nuclei. Fewer terminals were present in the peduncular nucleus, extreme dorsal lateral nucleus, ventral nucleus and lateral nucleus. Interestingly, most of the extreme dorsolateral, peduncular, ventral and lateral nuclear labeling was located at middle to inferior pontine levels (Figure 5).

### Contralateral Pontine Labeling From the LPMCv

The fiber pathway from LPMCv to the contralateral basis pontis followed a similar course as the LPMCd fibers. For example, as the labeled fibers passed across the midline, some abruptly terminated in the contralateral paramedian region, whereas the majority of fibers continued dorsally arching over and around the LPF and into the dorsomedial corner of the basis pontis gray matter (Figures 3F, 7a,b). Once within the dorsal tier nuclear region fibers passed laterally to innervate dorsally positioned pontine targets. Fewer labeled fibers passed the midline and arched ventrally to innervate the medial region of the ventral nucleus (Figure 7c).

In terms of terminal labeling, of the three motor cortical regions studied the LPMCv projection gave rise to a comparatively moderate cCPP (Figure 9). In general, contralateral labeled fibers terminated at middle and lower pontine levels. No contralateral projection was found at upper contralateral pontine levels (Figure 6). In terms of topography, the LPMCv cCPP targeted primarily the medial pontine region (Figures 6, 9). This included the paramedian nucleus, dorsomedial nucleus, medial part of the peduncular nucleus and medial part of the reticular nucleus. Labeled terminals were also found in the medial part of the ventral nucleus in case SDM57 and in the dorsolateral nucleus in case SDM72. Additional labeling involved the median nucleus in case SDM72.

## Discussion

**Figure 10:**
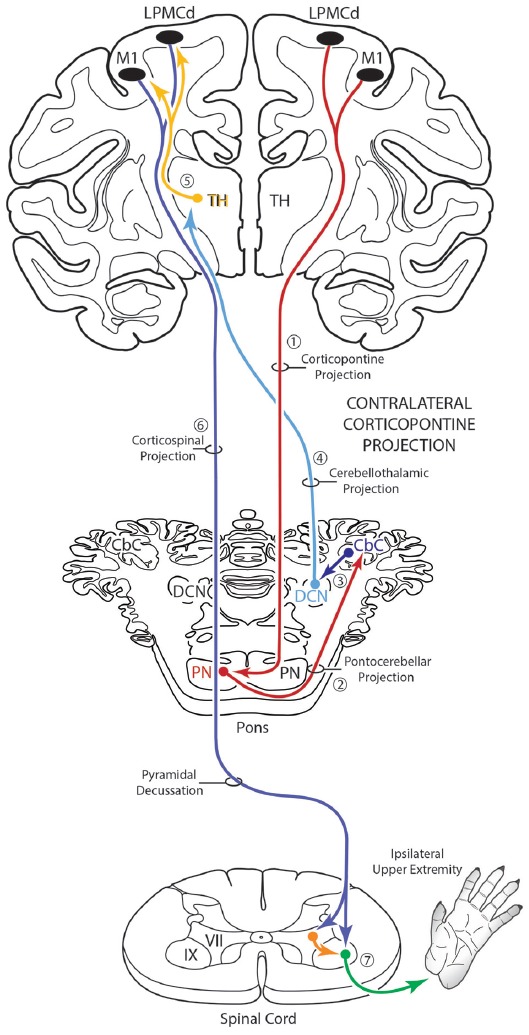
Schematic diagram of the classical motor cerebrocebellar system with the modification of the contralateral corticopontine projection (cCPP) in the first stage (1) of the circuit. Specifically, we found M1, LPMCd and LPMCv (not illustrated) all gave rise to a cCPP (1), demonstrating that the corticopontine projection from the lateral motor region is bilateral. Considering the sequential stages of the cerebrocebellar circuit as classically reported (stages 2–7, and as illustrated in Figure 1), the overall effect of the cCPP would theoretically be on the ipsilateral upper extremity. Thus, the CPP may play a role in both right and left upper limb movements.

The primate corticopontine projection is the largest descending cortical projection to the brainstem (Afifi and Bergman, 1980) and current understanding presents it as an exclusively ipsilateral axon projection system from the motor cortex to the basilar pontine gray matter (Figure 1; e.g., Dhanarajan et al., 1977; Carpenter, 1991; Kiernan et al., 2014; Siegel et al., 2015). However, one previous report in the non-human primate demonstrated labeled axon terminals from the precentral and lateral premotor cortices located along the midline and adjacent contralateral paramedian basilar pontine (Hartmann-von Monakow et al., 1981). Our observations not only validate the general finding of a primary motor and premotor cCPP (Figure 10) to midline and paramedian regions, but extend these observations to include the more laterally located nuclei of the basis pontis. In general, our observations agree with the previous report of the contralateral projection taking the form of scattered terminal patches (Hartmann-von Monakow et al., 1981; Figures 3B,C,F). Our findings also show that the M1 cCPP is relatively light, the LPMCv cCPP is moderate in comparison, and the LPMCd cCPP is the densest projection of the three motor areas studied, including being more widespread (Figure 9). It should be emphasized that the contralateral pontine projection from the precentral motor region is much lighter compared to its powerful ipsilateral counterpart (Figure 8A). However, from a comparative standpoint it must be recognized that the iCPP is the most powerful of all the descending cortical brainstem projection systems, so the relative percent of cCPP contributions (Figure 8B), can underestimate the potential impact of a relatively large number of contralateral boutons which were found in the present study to represent the cCPP, particularly from the lateral premotor cortical region. Furthermore, it is noteworthy to mention that the injection sites in the premotor region did not involve the total surface area of either the dorsal or ventral premotor regions. Thus, larger injection sites that involved more of the respective lateral premotor areas would likely result in a far greater number of cCPP labeled boutons than reported in the present study. Indeed, the corticopontine projection has been found to originate from the entire dorsal and ventral premotor fields (Glickstein et al., 1985).

### Anatomical Findings and Previous cCPP Observations

From the perspective of the monkey M1 arm/hand area, our findings verify the contralateral projection to the median nucleus and medial region of the reticular nucleus, but we did not observe a projection to the paramedian nucleus as reported by Hartmann-von Monakow et al. (1981). Our findings further demonstrate that the arm/hand region of M1 sends scattered projections to the full complement of dorsal tier nuclei including the dorsomedial, dorsal, dorsolateral and dorsolateral extreme nuclei (Figures 4, 9). With respect to the cCPP from LPMCd, our findings confirm the projection to the median nucleus, paramedian nucleus and medial part of the reticular nucleus (Hartmann-von Monakow et al., 1981). Our observations further demonstrate that all other contralateral PN (dorsomedial, dorsal, dorsolateral, dorsolateral extreme, peduncular, ventral and lateral) receive input from LPMCd (Figures 5, 9). Finally, we confirmed the findings reported by Hartmann-von Monakow et al. (1981) of axon terminals in the median nucleus, contralateral paramedian nucleus and contralateral dorsomedial nucleus from LPMCv. Our findings show that additional contralateral targets of the LPMCv projection include the reticular, peduncular, ventral and dorsolateral nuclei (Figures 6, 9). Although Brodal (1978a) mentions that large precentral motor cortex lesions gave rise to a small amount of contralateral pontine terminal degeneration, with small lesions of motor cortex resulting in no detectable contralateral terminal degeneration, it was not specified which anatomical locations contained the sparse labeling following the large lesions.

### cCPP Pathway Observations

We investigated the pathway by which the contralateral pontine projection gained access to the contralateral pontine gray matter since the design of current MRI—based tractography studies conducted *in vivo*, rely on tract tracing observations from experimental animals, and in the case of the human work extensive reliance is placed on non-human primate findings. Furthermore, we wanted to verify that the cCPP arose from descending fibers located within the ipsilateral LPF and was not a result of either transsynaptic labeling from ipsilateral pontine neurons or other possible routes including, for example, corpus callosum projections.

In agreement with the classical understanding of the descending projection to the basis pontis, we found descending axons in the midbrain crus cerebri to be exclusively ipsilateral to the origin of cortical tract tracer injection and positioned in the central (M1) and centromedial (LPMC) region of the crus cerebri as previously reported (Morecraft et al., 2007a). Indeed, one advantage of the current methodology was that we would be able to detect a single-labeled axon in the contralateral crus cerebri, but found none. In the pons, we also verified that labeling of vertically oriented fibers in the ipsilateral LPF was exclusively ipsilateral in all of the cases examined. Our observations further revealed that fibers initially leaving the ipsilateral LPF passed through the ipsilateral paramedian nucleus, ipsilateral dorsomedial nucleus and midline median nucleus to cross the midsagittal brainstem plane (Figures 7a–c). This clearly shows that the cCPP fibers cross the midline within the basis pontis. Once across the midline, cCPP fibers en route to the dorsal nuclei turn dorsally, and largely avoid traversing the contralateral LPF, then enter the medial most part of the dorsal nuclei (e.g., the dorsomedial nucleus; Figures 7a,b). From this point fibers travel laterally to reach other dorsal tier terminal targets (Figure 3F). In terms of the ventral route, after crossing the midline, fibers redirected their trajectory ventrally and avoid passing through the region of the contralateral LPF, then proceed to sweep laterally across the ventral region of the pontine gray matter (Figure 7c). Our findings suggest that these fiber pathways will be challenging to map *in vivo* in the human brain if a cCPP exists. Indeed, crossing fibers did not form a major fascicle, or appear as a coalesced fiber bundle. Instead, we found individually labeled fibers weaved their way through the ipsilateral paramedian, ipsilateral dorsomedial and median gray matter regions, and this pattern continued within the contralateral pontine gray matter (Figures 3C,F).

It has been commonly recognized that fibers destined for the contralateral pontine tegmentum emerge from the dorsal part of the ipsilateral LPF, enter the ipsilateral tegmentum, then cross the midline within the tegmentum proper (Figure 7d). However, our findings show that another axon pathway to the contralateral tegmental region exists, albeit more minor than the classically recognized route mentioned above. Specifically after crossing the midline through the basis pontis proper, some fibers that arched dorsally passed completely through the dorsal tier nuclear region to enter the contralateral tegmental region (Figures 7a,b).

### Potential Functional Implications

The functional implications of our cCPP observations are unclear but some speculation can be made based upon what is known about the classical cerebrocerebellar circuitry mediating upper limb motion via the classically recognized iCPP (Figure 1). Indeed, the corticopontine projection is the largest descending cortical brainstem pathway and represents the first stage of one of the most recognized multisynaptic circuits in the CNS, the cerebrocerebellar system (Brodal, 1978b; Schmahmann and Pandya, 1997b). In simple terms, it is well known that a massive M1/LPMC CPP innervates the ipsilateral pontine nuclei (iCPP) which in turn, project primarily to the contralateral cerebellar hemisphere via the contralateral middle cerebellar peduncle (Figure 1). Cerebellar output directed back to the cerebral cortex (from the deep cerebellar nuclei (DCN)) ascends in the superior cerebellar peduncle which crosses the midline to innervate nuclear targets in the contralateral thalamus. Finally, the thalamus projects to the ipsilateral motor cortex which, in higher order primates, controls primarily the opposite upper extremity (i.e., the contralateral limb) via a powerful and nearly exclusive CSP (Morecraft et al., 2013; Figure 1). The functional significance of this system has long been grounded in clinical and experimental observations demonstrating unilateral cerebellar lesions result in severely impaired movements in the upper limb positioned ipsilateral to the cerebellar injury (Holmes, 1917; Brooks et al., 1973).

Considering the overall laterality of the cerebrocerebellar system reviewed above, and assuming that all other components of the cerebrocebellar circuit would be identical to those of the iCPP (i.e., crossed cerebellar input, crossed cerebellar output, and an ipsilateral thalamocortical projection back to motor cortex), a cCPP in the first stage of the circuit would alter, or shift the overall effect of the pathway to the opposite hemisphere/motor cortex from which the cCPP originated. Theoretically, this would result in the cCPP affecting the upper extremity positioned on the same side (i.e., the ipsilateral limb) from which the cCPP originated (Figure 10). Overall, this would indicate that the total CPP from one motor cortex, in addition to having a dominant effect on the contralateral limb (via the iCPP), may have some, albeit more minor effect on control of the ipsilateral limb as well (via the cCPP). This pathway may be important for coordination of bimanual actions in which one hand performs the main action (e.g., rotating a jar lid) via the iCPP while the other performs a postural action (holding the jar) via the cCPP. Supporting this possibility include clinical observations showing that unilateral cerebellar stroke has bilateral effects on arm/hand movements with significant dysfunction of the hand ipsilateral to the cerebellar lesion as classically recognized, along with mild impairments to the arm/hand contralateral to the cerebellar lesion (e.g., Immisch et al., 2003; Anens et al., 2010).

For the above to be possible, one would assume that the cCPP from M1/LPMC should to some extent, overlap, or interdigitate with the iCPP from M1 (e.g., from the opposite hemisphere). This would seem highly probable given that significant terminal fiber overlap occurs amongst many descending corticopontine projection systems, although some topographic organization for each major descending projection can be discerned (Schmahmann, 2000). Indeed, when considering what has been reported for the iCPP from M1, some similarities with the cCPP from M1/LPMC can be recognized. For example, the heaviest iCPP from the M1 arm/hand area target pontine transverse levels IV–VIII (Schmahmann et al., 2004). In the current study, these levels were also the primary target for the cCPP form all three lateral precentral motor areas (Figures 4–6). At these levels, heavy iCPP from M1 labeling occurs medially as well as dorsally (Schmahmann et al., 2004—see Figures 7, 8) which was also the case for the collective cCPP from M1, LPMCd and LPMCv (Figures 4–6). Although sparse ventral nuclei cCPP labeling was only found from LPMCd in our study, the iCPP from M1 in case 5 of Schmahmann et al. (2004; which appears to be located in the lateral part of the M1 arm/hand area) appears to significantly involve the ventral and lateral nuclei. However, our M1 arm/hand injection sites were located at mid-levels of the M1 arm/hand representation (e.g., centered in the ICMS mapped arm/hand representation) which appears to be similar to the M1 arm/hand injection site location of Case 6 in the Schmahmann et al. (2004) report (see their Figure 8). In their Case 6, very little labeling appears to be located ventrally, and this was confined primarily to the ventral part of the peduncular nucleus. Interestingly, we also found scattered patches of labeling in the ventral region of the peduncular nucleus in all of our M1 and LPMC cases, although these labeled areas were very light (Figures 4–6, 9). Thus, it appears as if the cCPP from primary motor and lateral premotor cortices have some topographical characteristics that parallel the more powerful iCPP M1 projection, indicating that these projections systems may to some extent overlap with the cCPP providing some information to the ipsilateral limb cerebrocebellar circuit.

**Figure 11:**
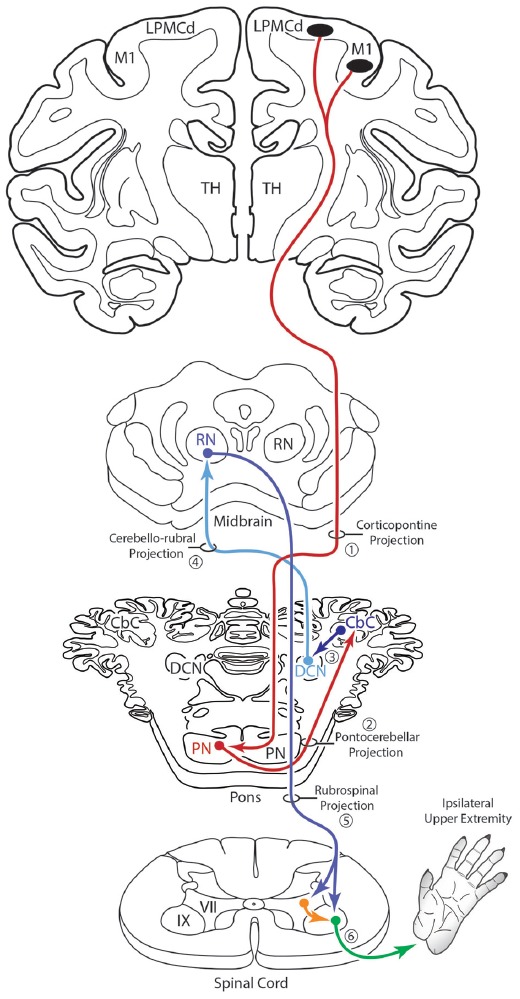
Summary diagram illustrating a potential contribution of the cCPP on the subcortical cerebellar circuit influencing the red nucleus which in turn projects to the contralateral spinal cord. The organization of the subcortical circuit positioned downstream on the cCPP is based upon the circuit characteristics of the classic cerebello-rubral (dentato-rubral) projection system.

An additional, subcortical circuit involving the cCPP that could also have implications for control of the ipsilateral limb would be through ascending cerebellar connections ending in the red nucleus (Figure 11). That is, instead of the ascending cerebello-thalamo-cortical pathway discussed above, one could consider ascending cerebellar output which targets the contralateral red nucleus (e.g., the cerebello-rubro circuit; Kennedy et al., 1986). The red nucleus in turn, sends a major projection to the contralateral spinal cord (Kuypers et al., 1962; Kennedy et al., 1986; Burman et al., 2000) and it is well known that this descending tract mediates distal upper extremity motor control (Lawrence and Kuypers, 1968; Belhaj-Saïf et al., 1998). Again, the net effect of this pathway would also be on the upper limb ipsilateral to the precentral cortical origin of the cCPP (Figure 11).

It is important to point out that there are other bilateral connections in the non-human primate cerebrocebellar circuit, which would supplement the powerful connections mediating contralateral limb movements, and potentially have an effect on ipsilateral limb movements. Brodal (1979) has shown bilateral pontocerebellar projections (with a contralateral predominance) to lobes IV, V and VI of the cerebellar cortex. Recent transneuronal transport application of neurotropic virus has shown that this region of the cerebellar cortex is part of the ascending loop linking the cerebellum to the M1 arm representation (Kelly and Strick, 2003). Similarly, following injections of WGA-HRP into the M1 arm area, transneuronal transport of the WGA-HRP tracer resulted in bilateral neuronal labeling in the dentate and interposed cerebellar nuclei, with significant contralateral predominance (Wiesendanger and Wiesendanger, 1985). This finding has been recently verified using retrograde transneuronal transport of rabies virus to identify cerebellar nuclei that project to the arm area of M1 (Lu et al., 2012). Specifically, it was reported by Lu et al. (2012) that “most” of the retrograde labeling in the DCN (dentate and interposed) was found contralateral to the cortical injection site, indicating that some ipsilateral DCN contained retrograde viral labeling. Immisch et al. (2003) have summarized some physiological observations that support a functional role for these nonconventional anatomical findings. Collectively, these observations suggest that some ipsilateral limb processing may occur at multiple levels of the cerebrocebellar system.

### Additional Clinical Considerations

**Figure 12:**
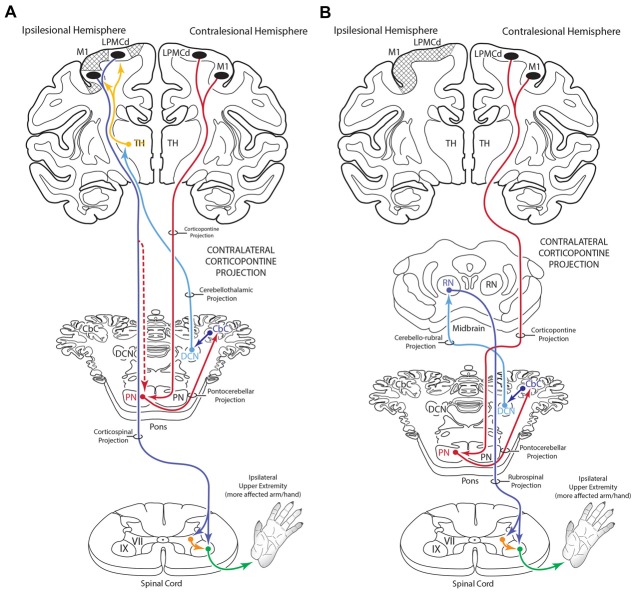
Summary diagram **(A)** illustrating the potential influence of the cCPP from the non-lesioned hemisphere on the recovery process of the more affected limb following subtotal unilateral motor cortex injury. Following partial motor cortex destruction, a cCPP may augment the effects of the surviving components of the iCPP (red dashed line) originating from the lesioned hemisphere. Also illustrated **(B)** is the potential parallel contribution of a cCPP to motor recovery of the more affected limb through the rubrospinal projection, particularly following massive unilateral motor cortex injury. In both illustrations the cross hatching represents lesioned cortex.

Understanding the role of the non-lesioned hemisphere following upper extremity motor paresis is essential for improving recovery outcome. Although the effects of corticocortical interconnections, and specifically, the influence of callosal disinhibition on the non-lesioned hemisphere has received considerable attention, examining the contribution of descending projections from the non-lesioned hemisphere represents another important area of consideration. Indeed, following injury to M1 and the adjacent LPMC in the non-human primate, we recently found the CSP from contralesional M1 enhances its terminal projection to spinal laminae controlling axial/proximal upper extremity movements (Morecraft et al., 2016). Based upon the current findings, a potential cCPP arising from the contralesional hemisphere could, by anatomical design of the cerebrocebellar system, have some influence on the motor recovery process of the “more affected” limb following subtotal unilateral precentral cortex injury. This may occur through the ascending cerebello-thalamo-cortical projection to spared regions of the ipsilesional precentral motor cortex following subtotal precentral injury (Figure 12A), as well as through ascending cerebellar output that targets the red nucleus following subtotal injury (Figure 12B). Indeed, it is possible that the cCPP, by influencing red nucleus descending projections, could play some role in recovery of the more affected hand following extensive unilateral precentral motor cortex injury (Figure 12B). If present in the human brain, a potential iCPP may also contribute to functional activation occurring in the contralesional precentral/premotor cortical region when a patient uses the more affected arm/hand for performing a motor task (e.g., Chollet et al., 1991; Weiller et al., 1992; Johansen-Berg et al., 2002; Ward et al., 2003, 2007; Nair et al., 2007; Bestmann et al., 2010; Rehme et al., 2011). Our finding of a bilateral CPP and specifically a cCPP from motor cortex may be important for interpreting cerebellar hemispheric activation patterns following unilateral motor cortex injury. For example, it is possible that activation of contralesional M1 through the cCPP may contribute to increases in contralesional cerebellar hemisphere activation (hemisphere on the side of the more affected arm/hand) when the more affected hand is used (Figure 12A; e.g., Small et al., 2002). Importantly, such increases in cerebellar activation were correlated with recovery of fine hand motor function over 6 months post-stroke in patients who recovered good hand function (Small et al., 2002).

### Technical Considerations

The design-based stereological approach that was used in this study to estimate total number of biological particles (terminal axon boutons) included the application of the 3-D optical fractionator probe applied within a known volume of a defined region of interest (ROI; each nuclei). In short, unbiased sampling was applied such that each location along the tissue section axis had an equal probability of being included in the sample and all locations in the plane of section (excluding the set guard zones) had an equal probability of being sampled with the probe (e.g., Gundersen, 1986; West et al., 1991; for review, see Napper, 2018). Counting rules were also applied across all case material so that all boutons had equal probabilities of being counted. Importantly, according to experts in the stereology field, the use of a 3-D probe avoids sampling biases and the most important feature of these probes is that they are not affected by variations in size, shape, orientation and distribution of the biological structures/particles of interest (e.g., West, 2012; Olesen et al., 2017). Therefore, the fact that the iCCP was significantly larger and occasionally formed large lamella like shapes, vs. the more diffuse/scattered labeling and lack of lamellar formation on the contralateral side as noted here should not have affected the estimation process of total bouton number. It is also noteworthy to mention that the iCPP lamellar patches typically extend across multiple nuclei (Brodal, 1978b; Hartmann-von Monakow et al., 1981; Brodal and Bjaalie, 1997), thus are not confined to a given nucleus. Our ROI’s were not affected by these terminal patterns and shapes as each ROI was based upon an individually defined nuclear boundary with a known/estimated volume. In addition, all basilar PN (ipsilateral, contralateral and median) were included in our analysis. Finally, we would like to point out that the applied stereological approach and probe (including counting brick dimensions and brick spacing intervals) have been shown to appropriately estimate the total number of terminal boutons in well-defined ROI’s that receive a dense and compact corticospinal and corticoreticular projection, as well as a light and dispersed corticospinal and corticoreticular projection (McNeal et al., 2010; Morecraft et al., 2015a, 2016; Darling et al., 2018).

## Conclusion

In summary, we have shown that a cCPP arises from M1, LPMCv and LPMCd demonstrating that the corticopontine projection from the lateral precentral motor region is bilateral. The M1 cCPP targets the dorsal basis pontis region and the LPMCv projection the medial basis pontis region. The LPMCd cCPP is widespread, involving all contralateral PN, with its concentration of terminals ending in the medial and dorsal regions. These findings indicate that the precentral CPP from one hemisphere, in addition to having a dominant effect on the contralateral limb (via the powerful iCPP), may have some, albeit smaller, effect on cerebrocebellar mechanisms affecting the ipsilateral limb as well (via the cCPP). Based upon the anatomy, it is also possible that the cCPP influences the motor recovery process of the more affected limb located ipsilateral to the cortical origin of the cCPP, and this may contribute to functional activation observed in the contralesional cortical hemisphere when severely injured patients use their more affected upper limb for reaching and grasping.

## Author Contributions

All authors contributed to the elaboration of the article that led to preparation of the manuscript. All authors had full access to all of the data in the study and take responsibility for the integrity of the data and the accuracy of the data. RM: study concept and design; study supervision. RM, JG and KS-M: development of data. JG, KS-M and RM: acquisition of data. RM, WD, MP, JG, KS-M and DR: interpretation of data. RM, KS-M, JG, WD, MP and DR: drafting of manuscript.

## Conflict of Interest Statement

The authors declare that the research was conducted in the absence of any commercial or financial relationships that could be construed as a potential conflict of interest.

## Abbreviations

BDA: biotinylated dextran amine
cCPP: contralateral corticopontine projection
iCPP: ipsilateral corticopontine projection
FD: fluorescein dextran
LPMCd: dorsolateral premotor cortex
LPMCv: ventrolateral premotor cortex
LYD: lucifer yellow dextran
M1: primary motor cortex.
